# Mir-331-3p Inhibits PRRSV-2 Replication and Lung Injury by Targeting PRRSV-2 ORF1b and Porcine *TNF-*α

**DOI:** 10.3389/fimmu.2020.547144

**Published:** 2020-09-25

**Authors:** Xiangbin You, Yilin Qu, Yue Zhang, Jingshu Huang, Xiaoxiao Gao, Chengyu Huang, Gan Luo, Qian Liu, Min Liu, Dequan Xu

**Affiliations:** ^1^Key Laboratory of Swine Genetics and Breeding of Ministry of Agriculture and Rural Affairs, Huazhong Agricultural University, Wuhan, China; ^2^Key Laboratory of Agricultural Animal Genetics, Breeding and Reproduction of Ministry of Education, Huazhong Agricultural University, Wuhan, China; ^3^Colleges of Animal Science and Veterinary Medicine, Huazhong Agricultural University, Wuhan, China; ^4^Agricultural Development Center of Hubei Province, Wuhan, China

**Keywords:** miR-331-3p, miR-210, lung injury, PRRSV, TNF-α, STAT1, ORF1b

## Abstract

Porcine reproductive and respiratory syndrome (PRRS) caused by a single-stranded RNA virus (PRRSV) is a highly infectious respiratory disease and leads to huge economic losses to the swine industry worldwide. To investigate the role of miRNAs in the infection and lung injury induced by PRRSV, the differentially expressed miRNAs (DE-miRs) were isolated from PRRSV-2 infected/mock-infected PAMs of Meishan, Landrace, Pietrain, and Qingping pigs at 9, 36, and 60 hpi. Mir-331-3p was the only common DE-miR in each set of miRNA expression profile at 36 hpi. Mir-210 was one of 7 common DE-miRs between PRRSV infected and mock-infected PAMs of Meishan, Pietrain, and Qingping pigs at 60 hpi. Mir-331-3p/mir-210 could target PRRSV-2 ORF1b, bind and downregulate porcine *TNF-*α/*STAT1* expression, and inhibit PRRSV-2 replication, respectively. Furthermore, *STAT1* and *TNF-*α could mediate the transcriptional activation of *MCP-1, VCAM-1*, and *ICAM-1*. *STAT1* could also upregulate the expression of *TNF-*α by binding to its promoter region. *In vivo*, pEGFP-N1-mir-331-3p could significantly reduce viral replication and pathological changes in PRRSV-2 infected piglets. Taken together, Mir-331-3p/mir-210 have significant roles in the infection and lung injury caused by PRRSV-2, and they may be promising therapeutic targets for PRRS and lung injury/inflammation.

## Introduction

Porcine reproductive and respiratory syndrome (PRRS) is characterized by severe reproductive failure and respiratory distress in pigs (1, 2). The disease was first reported in the late 1980s in the United States and first found in Beijing, China, in 1995 (3–6). At present, there are still many kinds of PRRS virus (PRRSV) sublineages in China, which poses a great threat to swine industry (7, 8).

PRRS is caused by a small, enveloped positive-sense, single-stranded RNA virus, PRRSV, which belongs to the family arteriviridae. The PRRSV genome is ~15 kb in length and contains at least 10 open reading frames (ORFs) (9). Among these ORFs, ORF1a and ORF1b account for ~75% of the viral genome encoding the proteins with apparent replicase and polymerase activity. It has been reported that a combination of ORF1a and ORF1b is essential for viral minus-strand RNA synthesis (10). ORF1b is directly related to the fatal virulence and replication efficiency of HP-PRRSV both *in vitro* and *in vivo* (11, 12).

The infection of PRRSV can be divided into three different stages: acute phase, persistent infection phase, and regression phase. In the acute infection phase, the lung is the main organ of the PRRSV invasion. Porcine alveolar macrophages (PAMs) are the primary target cells of PRRSV and the main viral replication sites during infection (13, 14). PAMs are also the first line of defense against pathogenic microbes during pathogen invasion. Previous reports showed that highly pathogenic PRRSV resulted in lung injury of the infected pigs (15). In addition, PRRSV can survive for over 250 days in the host after infection (16). Sustained infection of PRRSV can lead to lung injury, mainly manifesting as diffuse inflammatory damage, and eventually result in a series of inflammation cascade reactions (17, 18). Lung injury can cause destruction of the immune system, which in turn can lead to death from co-infection (19).

MiRNAs (microRNAs) are non-coding RNAs with 18–23 nucleotides and target specific genes by binding to ORFs or 3′untranslated region (UTRs) of RNA to perform their functions (20, 21). MiRNAs play important roles in biological processes such as cell proliferation, differentiation, apoptosis, metabolism, and immunity (22, 23). There is increasing evidence that miRNAs are involved in virus-mediated lung injury (24, 25). Previous study has reported that mir-27-3p played an important role in LPS-induced acute lung injury (ALI) (26). Mir-30b-5p played an important role in the inflammation of ALI and might be an important therapeutic target for ALI (27).

In the present study, the differentially expressed miRNAs (DE-miRs) were isolated from PRRSV-2 infected/mock-infected PAMs of Meishan, Landrace, Pietrain, and Qingping pigs at 9 h post infection (hpi), 36, and 60 hpi. Mir-331-3p was the only common DE-miR between PRRSV infected and mock-infected PAMs of all 4 pig breeds at 36 hpi. Mir-331-3p was also one of 4 common DE-miRs between PRRSV infected and mock-infected PAMs of Meishan, Pietrain, and Landrace pigs at 60 hpi. Mir-210 was one of 7 common DE-miRs between PRRSV infected and mock-infected PAMs of Meishan, Pietrain, and Qingping pigs at 60 hpi. Mir-331-3p and mir-210 were predicted to target ORF1b, which was confirmed by double fluorescence reporter assay. Mir-210 and mir-331-3p could inhibit the replication of PRRSV-2 and were involved in the regulation of lung injury by targeting *STAT*1 and *TNF-*α, respectively. The pEGFP-N1-mir-331-3p could significantly reduce viral replication and attenuate lung injury in PRRSV-2 infected piglets *in vivo*. The regulation mechanisms of mir-331-3p and mir-210 were analyzed in the infection and lung injury caused by PRRSV-2.

## Materials and Methods

### Ethics Statement

All animal procedures were approved by the Scientific Ethic Committee of Huazhong Agricultural University, Wuhan, China.

### Cell Line and Virus

Marc-145 cells were obtained from China Center for Type Culture Collection (CCTCC) and cultured in RPMI 1640 (Hyclone, Logan, UT, USA) supplemented with 10% fetal bovine serum (CLARK Bioscience, Virginia, USA) in an incubator at 37°C with 5% CO_2_. PRRSV-2 strain WuH3 (GenBank accession No.HM853673) was kindly provided by Dr. Xiao Shaobo.

### DE-miRs Analyses

PAMs were isolated from Pietrain (P), Qingping (QP), Meishan (MS), and Landrace (L) pig breeds by bronchoalveolar lavage under aseptic conditions (28, 29). Bronchoalveolar lavage was performed using pre-chilled PBS containing 200 μg of penicillin and 200 U of streptomycin per mL, after which bronchoalveolar lavage cells were collected, filtered, and centrifuged. PAMs were washed three times with PBS, after which they were suspended and cultured in RFMI 1640 medium with 10% FBS containing 100 μg of penicillin and 100 U of streptomycin per mL. After incubation for 2 h at 37°C, the culture medium was changed to further purify PAMs by plastic adherence of PAMs on cell culture flasks. Then, the PAMs of 5 pigs of each breed were infected with PRRSV-2 strain WuH3 at multiplicity of infection (MOI) of 0.1 PFU/cell. The PRRSV-2 infected PAMs were collected at 9, 36, and 60 hpi and mixed evenly. The control group (mock-infected) PAMs were infected with culture medium and collected at 9, 36, and 60 h. Total cellular RNA was isolated using the Trizol reagent (Invitrogen, Cashman, CA, USA) to analyze the differential expression of miRNAs. Deep sequencing was performed by the Illumina/solexa Genome Analyzer (BGI, Shenzhen, China). Twenty-four miRNA libraries were constructed. The expression of miRNAs was normalized and analyzed by calculating fold-change and *p*-value (30, 31). A miRNA was labeled as differentially expressed, when |log2(fold change)| ≥ 1 and *p* ≤ 0.01.

### Bioinformatics and Luciferase Reporter Assay

JASPAR software was used to analyze the *TNF-*α 5′ flanking sequence. TargetScan, miRbase, RNAhybrid, and ViTa software were used to predict target genes of miRNAs. The 3′UTR of *TNF-*α containing putative mir-331-3p binding site and the 3′UTR of *STAT1* containing putative mir-210 binding site were amplified by PCR and cloned into pmirGLO vector (Promega, Madison, Wisconsin, USA), respectively. Mutation sites were identified in the predicted target sites of mir-331-3p/mir-210 in the 3′UTR of *TNF-*α/*STAT1*. The ORF1b sequence containing the mir-331-3p binding site and the ORF1b sequence containing the mir-210 binding site were amplified, respectively. Subsequently, these two amplified sequences were connected to the pmirGLO vector and named ORF1b-331-WT and ORF1b-210-WT, respectively. Fusion PCR was used to construct the binding site-specific mutant plasmids, and the two resultant plasmids were named ORF1b-331-MUT and ORF1b-210-MUT, respectively. The primer sequences for plasmid construction were listed in Supplementary Table 1. For luciferase reporter assay, mir-331-3p or mir-210 were co-transfected with the corresponding dual-fluorescence reporter plasmid into Marc-145 cells in 24-well plates by using Lipofectamine 2000™ (Invitrogen, Cashman, CA, USA). At 48 h post-transfection, the dual-luciferase reporter assay system (Promega, Madison, Wisconsin, USA) was used to measure the luciferase activity.

### Cell Transfection and Viral Infection

Marc-145 cells were seeded into 6-well plate and cultured at 37°C in humidified 5% CO_2_ atmosphere. After 24 h incubation, miRNA or siRNA were transfected into cells of each well by using lipofectamine 2000 (Invitrogen, Cashman, CA, USA). The sequences of miRNA mimics/inhibitors and siRNA were listed in Supplementary Table 2. After 5 h, 3% of the cell maintenance medium was added, and the normal growth medium was added after 1 h PRRSV-2 infection at MOI = 0.1. The cells were collected for the extraction of total protein and total RNA at 36 h post viral infection.

### RNA Extraction, Reverse Transcription, and qRT-PCR

Total RNA was extracted from tissues or cells with TRIzol reagent (Invitrogen, Cashman, CA, USA). RNA (500 ng) was reversely transcribed with RevertAid First Strand cDNA Synthesis Kit (Thermo Fisher Scientific, Waltham, MA, USA). Quantitative real-time PCR (qRT-PCR) assay was performed by using the SYBR Green real-time PCR Master Mix regents in the Roche LightCyler 480 system (Roche, Mannheim, Germany) according to the manufacturer's protocol. Primers used for qRT-PCR were shown in Supplementary Table 3. U6 or β-actin was applied as the internal control, while the fold changes were calculated by 2^−ΔΔCt^ method. Absolute quantification was used to detect the PRRSV copy number, and the primer ORF7-F/R and ORF7-probe were shown in Supplementary Table 3. All experiments were performed at least three times in triplicate.

### Western Blot Analysis

RIPA lysis buffer (Beyotime, Shanghai, China) was used to extract total protein. After SDS-PAGE, the protein was transferred to a polyvinylidene difluoride (PVDF) membrane (Millipore, Billerica, MA, USA), and then this membrane was blocked with skim milk for 2 h, followed by incubation at 4°C overnight with the following primary antibodies including anti-STAT1 (D120084-0025, Sangon Biotech, Shanghai, China), anti-TNF-α (A0277, ABclonal, Wuhan, China), anti-PRRS virus Nucleocapsid (4269, GeneTex, Alton Pkwy Irvine, CA, USA), anti-β-tubulin (GB11017B, Servicebio, Wuhan, China), and anti-β-actin (AC026, ABclonal, Wuhan, China), respectively. After incubation, PVDF membrane was washed with Tris-buffered saline Tween 20 (TBST) for three times, and then this membrane was incubated with secondary HRP-conjugated antibodies (G1213, Servicebio, Wuhan, China) and the protein on this membrane was visualized by using the ECL (enhance chemiluminescence) (Servicebio, Wuhan, China) in Western Blotting Detection System.

### Plasmids Construction

The pEGFP-N1-mir-331-3p plasmids were constructed by inserting mir-331-3p precursor into the pEGFP-N1 vector (Clontech, Mountain View, CA, USA). The porcine TNF-α 5′-flanking genomic region was amplified. Subsequently, the fragments were digested and inserted into pGL3-Basic vector (Promega, Madison, WI, USA). The construction vector primers were shown in Supplementary Table 1.

### Animals

Six, 4-week-old piglets were randomly selected and divided into two groups, pEGFP-N1-mir-331-3p treatment group and control group. The pEGFP-N1-mir-331-3p or pEGFP-N1 (at the dose of 2.5 mg/kg body weight) were mixed with D5W solution to finally obtain 3 mL mixture solution, respectively. This 3 mL solution was administered to piglets through intramuscular injection. At 5 h post intramuscular injection, the 1.5 mL of PRRSV-2 strain WuH3 (10^5.2^ TCID50) was administered to piglets. The rectal temperature of piglets was measured twice a day. On day 14, we performed pathological dissection and collected all the lungs and PAMs of the piglets. The sacrificed pigs were taken out and the animal experiments were carried out by random and blind method, *in vivo* experiment, three replicates for each of the two groups. All experiments were performed at least three times in triplicate, excluding average rectal temperatures.

### Histological Assay

After being fixed in 4% paraformaldehyde, lung tissues were embedded in paraffin. Lung tissues were analyzed by Hematoxylin-Eosin staining (H&E). The experiments followed the procedures previously reported (32). Finally, these sections were observed under an optical microscope (Olympus, Tokyo, Japan) to detect morphological changes of lung tissues. To further detect the expression of TNF-α, immunohistochemistry (IHC) experiments were performed using specific polyclonal anti-TNF-α (A0277, ABclonal, Wuhan, China). Briefly, tissue blocks were cut into sections. The sections were deparaffinized and dehydrated through xylene and graded alcohols, and then these sections were rehydrated with demineralized water. The sections were blocked with 3% hydrogen peroxide for 30 min, boiled in a 0.01 M sodium citrate buffer for 10 min at high temperature, and boiled for 10 min at low setting to expose the antigen, cooled naturally, and washed 3 times with PBS for 3 min each time. Then, the sections were blocked with goat serum at room temperature for 20 min. Primary antibody was incubated overnight at 4°C. The sections were washed with PBS and incubated with secondary HRP-conjugated antibodies (Servicebio, Wuhan, China) for 30 min. Diaminobenzidine staining and hematoxylin staining were performed. Differentiation was conducted with 1% hydrochloric acerbic for 30 s. The sections were then dehydrated with ethanol series, washed in xylene, embedded in paraffin wax, and photographed with a microscope (Olympus, Tokyo, Japan).

### Statistical Analysis

All experiments were performed at least three times in triplicate. The differences were assessed using two-tailed *t*-test or one-way ANOVA *in vitro* experiment. Non-parametric Mann-Whitney Statistical test was used *in vivo* experiment due to the few number of animals available (33, 34). Data were presented as mean ± SD. Statistically significant difference was presented at the level of ^*^p < 0.05 and ^**^p < 0.01. All the histograms and graphs were generated with GraphPad Prism version 5.0 and Adobe Photoshop CS5 software, respectively.

## Results

### Mir-331-3p and Mir-210 Were Differentially Expressed Between PRRSV-Infected and Mock-Infected PAMs

**Figure 1:**
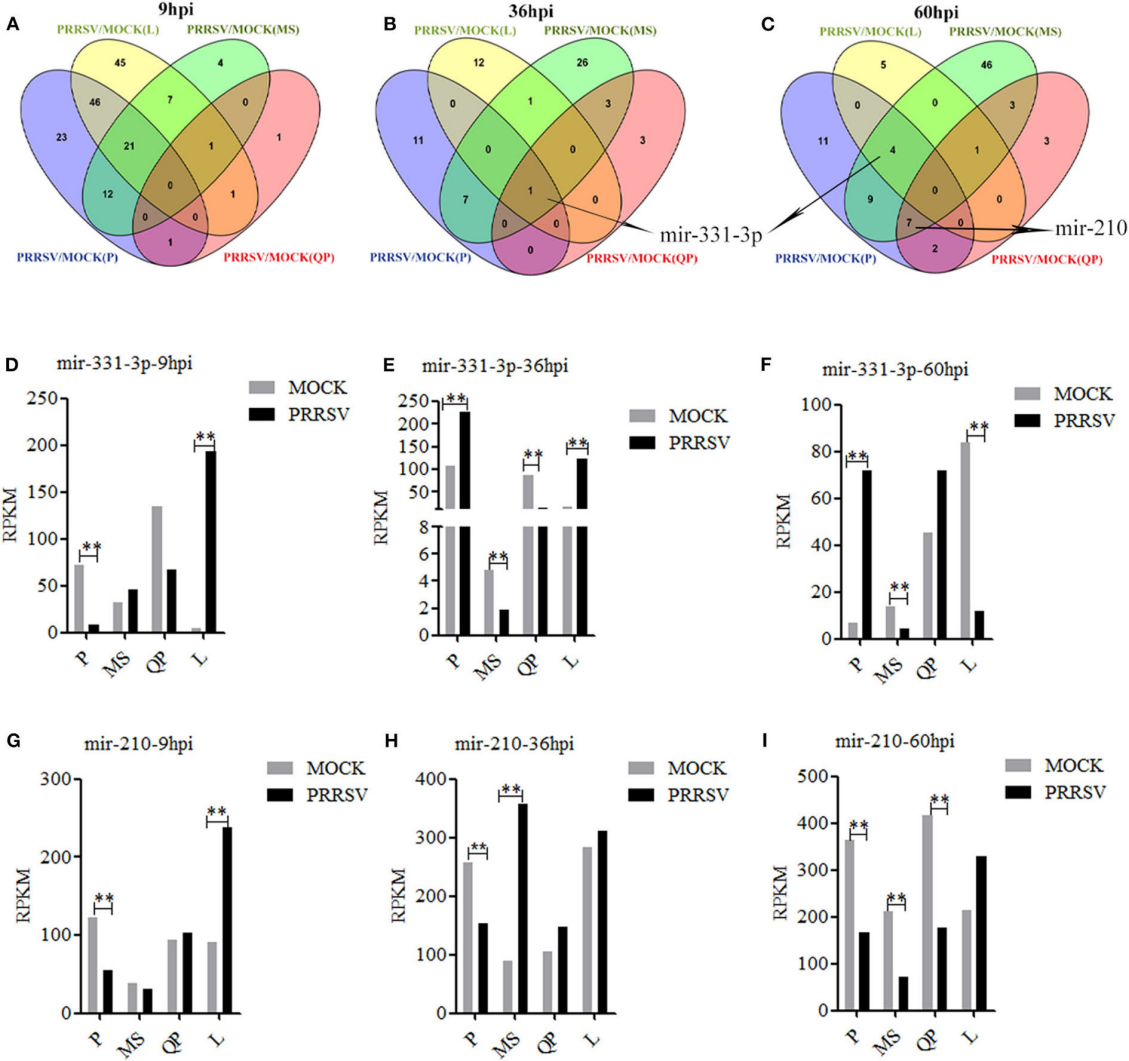
DE-miRs in PRRSV-infected/mock-infected PAMs from 4 pig breeds. Venn diagrams of DE-miRs between PRRSV-infected and mock-infected PAMs of Pietrain (P), Landrace (L), Qingping (QP), and Meishan (MS) pigs at 9, 36, and 60 hpi **(A–C)**. Mir-331-3p expression levels were analyzed in miRNA-sequencing data of PRRSV-infected/mock-infected PAMs from 4 breeds of pigs at 9, 36, and 60 hpi **(D–F)**. Mir-210 expression levels were analyzed in miRNA-sequencing data of PRRSV-infected/mock-infected PAMs from 4 breeds of pigs at 9, 36, and 60 hpi **(G–I)**. All values represent the mean ± s.d. of three independent experiments. **p < 0.01.

DE-miRs were analyzed using previously reported methods (30, 35). Four sets of miRNA expression profile were synthesized by Venn diagram analysis (Figures 1A–C). The Venn diagrams showed there were the most overlapped DE-miRs (67) between Pietrain and Landrace pigs in the early stage of PRRSV infection (9 hpi). There were the most overlapped DE-miRs (8 and 20) between Pietrain and Meishan pigs at 36 and 60 hpi. Among them, only mir-331-3p was significantly differentially expressed in each set of miRNA expression profile at 36 hpi (Figure 1B), and mir-331-3p was also one of 4 common DE-miRs between PRRSV infected and mock-infected PAMs of Meishan, Pietrain, and Landrace pigs at 60 hpi (Figure 1C). In addition, mir-210 was one of 7 common DE-miRs between PRRSV infected and mock-infected PAMs of Meishan, Pietrain, and Qingping pigs at 60 hpi. Mir-331-3p and mir-210 were both the common DE-miRs between PRRSV infected and mock-infected PAMs of Pietrain and Landrace pigs at 9 hpi (Figures 1D–I). These results implied that mir-331-3p and mir-210 might contribute to specific responses to PRRSV infection.

### Mir-331-3p and Mir-210 Directly Targeted PRRSV-2 ORF1b

**Figure 2:**
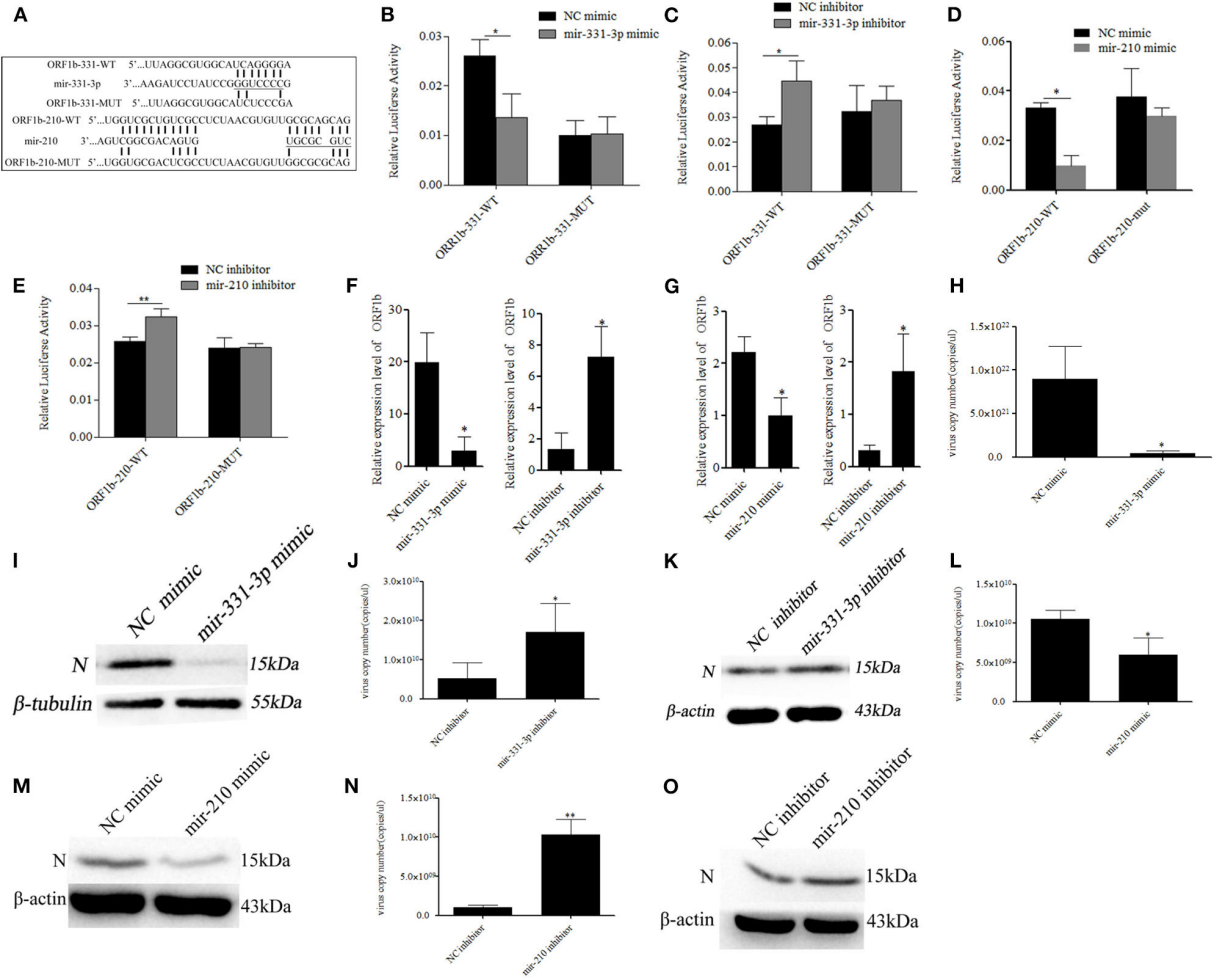
Mir-331-3p and mir-210 inhibit PRRSV-2 replication. Bioinformatical predication showed that ORF1b was a putative target gene of mir-331-3p and mir-210 **(A)**. The dual luciferase reporter assay was verify the bind of mir-331-3p and ORF1b in cells co-transfected with ORF1b-331-WT/ORF1b-331-MUT and mir-331-3p mimics **(B)** or mir-331-3p inhibitor **(C)**. The dual luciferase reporter assay was used to verify the bind of mir-210 and ORF1b in cells co-transfected with ORF1b-210-WT/ORF1b-210-MUT and mir-210 mimics **(D)** or mir-210 inhibitor **(E)**. ORF1b expression was quantitatively analyzed in Marc-145 cells infected with PRRSV-2 after transfection with mimics/inhibitor of mir-331-3p **(F)** or mir-210 **(G)**. Marc-145 cells were transfected separately with mir-331-3p mimics and mir-331-3p inhibitor, and then infected with PRRSV-2 (MOI = 0.1). The cells were harvested at 36 h post PRRSV-2 infection, and qRT-PCR **(H,J)**, and western blot **(I,K)** was carried out to detect PRRSV-2 replication. Meanwhile, Marc-145 cells were transfected separately with mir-210 mimics and mir-210 inhibitor, and then infected with PRRSV-2 (MOI = 0.1). The cells were harvested at 36 h post PRRSV-2 infection, and qRT-PCR **(L,N)** and western blot **(M,O)** was carried out to detect PRRSV-2 replication. All values represent the mean ± s.d. of three independent experiments.*p < 0.05, **p < 0.01.

The RNAhybrid and ViTa softwares were used to predict the target sites of mir-331-3p and mir-210 in PRRSV genome. The results indicated that mir-331-3p and mir-210 could target in 9,850 to 9,856 and 9,953 to 9,961 bp of PRRSV-2 ORF1b through seed pairing, respectively (Figure 2A). We aligned the target sequences in 15 representative PRRSV strains and found that mir-331-3p and mir-210 could target the ORF1b of PRRSV-2 strains (Supplementary Figure 1) and not target PRRSV type 1 strains. Further, luciferase reporter analysis was used to verify the binding of mir-331-3p and ORF1b. The ORF1b-331-WT luciferase reporter plasmid was co-transfected with mir-331-3p mimics or negative control (NC) into Marc-145 cells, and luciferase activity was found to be significantly suppressed by mir-331-3p mimics (*p* = 0.011). However, mir-331-3p had no effect on ORF1b-331-MUT luciferase reporter plasmid (Figure 2B). Similar results were obtained when ORF1b-331-WT (*p* = 0.019) or ORF1b-331-MUT (*p* = 0.29) was co-transfected with mir-331-3p inhibitors into Marc-145 cells (Figure 2C). In addition, the luciferase activity of ORF1b-210-WT transfected with mir-210 mimics was significantly lower than that transfected with NC mimics (*p* = 0.04) (Figure 2D). Mir-210 inhibitor significantly promoted the luciferase activity of ORF1b-210-WT (*p* = 0.003), but not that of ORF1b-210-MUT (*p* = 0.98) (Figure 2E). The qRT-PCR assay showed that mir-210 (*p* = 0.014, *p* = 0.03, Figure 2F) and mir-331-3p (*p* = 0.03, *p* = 0.05, Figure 2G) could significantly inhibit the expression of ORF1b. Thus, it could be concluded that mir-331-3p and mir-210 could directly target the PRRSV-2 genome.

To investigate whether mir-331-3p and mir-210 inhibit PRRSV-2 replication by binding to ORF1b, Marc-145 cells were transfected with the mimics or inhibitor of each miRNA (10 nM), followed by infection with PRRSV-2 strain WuH3 at an MOI of 0.1. The results showed that overexpression of mir-331-3p significantly inhibited PRRSV-2 copy number (*p* = 0.017, Figure 2H). Meanwhile, a similar tendency was observed at N protein (PRRSV-2 ORF7 encoded) level (Figure 2I). Additionally, the inhibition of mir-331-3p led to the significantly increased the number of PRRSV-2 copies (*p* = 0.03, Figure 2J) and expression level of N protein (Figure 2K) in Marc-145 cells. In addition, mir-210 also significantly inhibited PRRSV-2 replication and N protein expression (*p* = 0.02, *p* = 0.008, Figures 2L–O). The above-mentioned findings provided evidence that mir-331-3p and mir-210 could inhibit the viral replication by targeting PRRSV-2 genome.

### Mir-331-3p Negatively Regulated *TNF-α*

**Figure 3:**
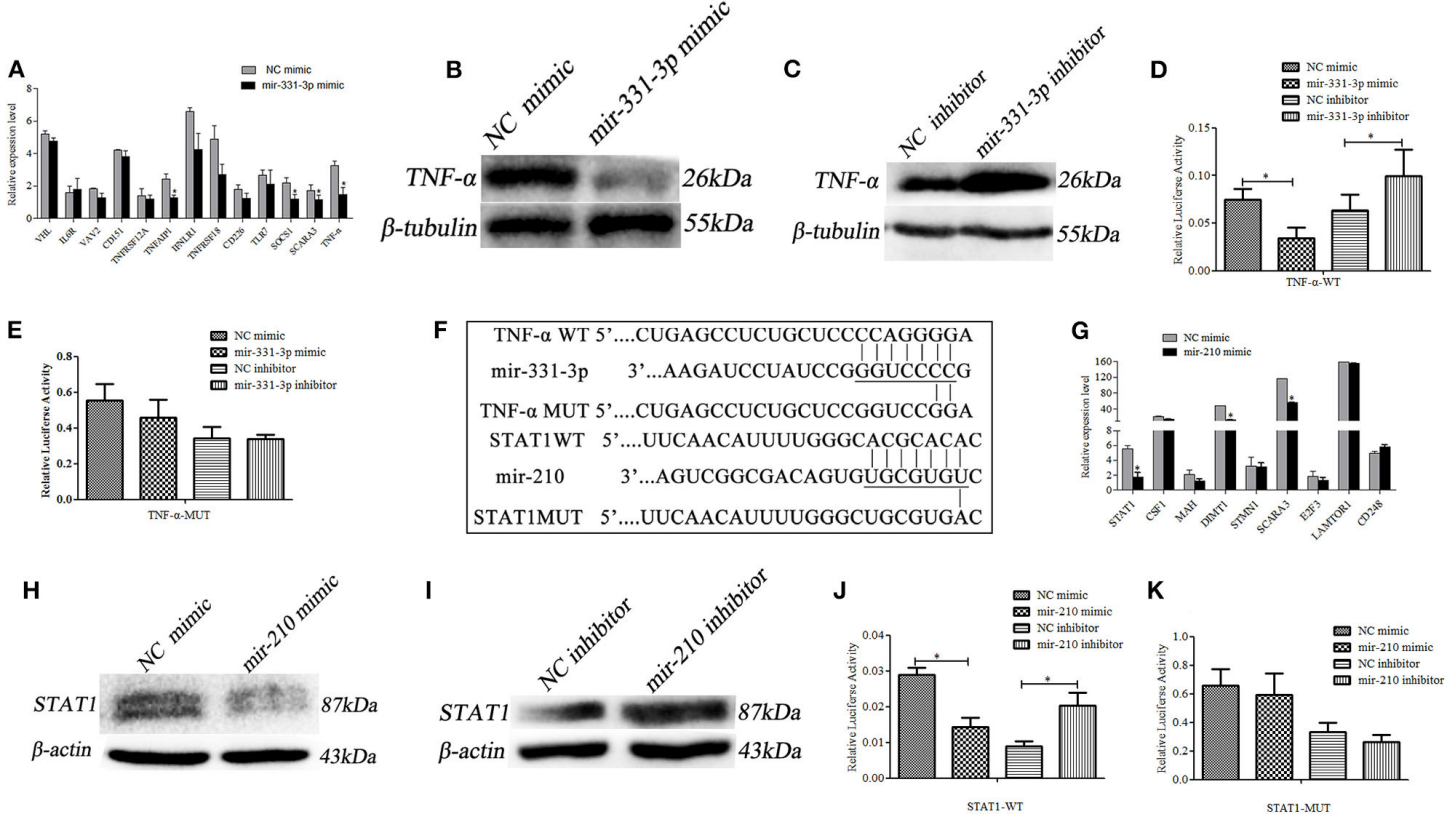
Mir-331-3p and mir-210 regulate target genes *TNF-*α and *STAT*1, respectively. Marc-145 cells were transfected with mir-331-3p to detect the expression of the potential target genes **(A)**. TNF-α expression was detected by western blot in Marc-145 cells transfected with mir-331-3p mimics **(B)** or mir-331-3p inhibitor **(C)**. The dual luciferase reporter assay was used to verify the bind of mir-331-3p and TNF-α in cells co-transfected with mir-331-3p mimics/inhibitor and TNF-α-WT **(D)** or TNF-α-MUT **(E)**. Bioinformatical predication showed that TNF-α and STAT1 were the target gene of mir-331-3p and mir-210, respectively **(F)**. Marc-145 cells were transfected with mir-210 mimics to detect the expression of the potential target genes **(G)**. STAT1 protein expression was detected by western blot in Marc-145 cells transfected with mir-210 mimics **(H)** or mir-210 inhibitor **(I)**. The dual luciferase reporter assay was used to verify the bind of mir-210 and STAT1 in cells co-transfected with mir-210 mimics/inhibitor and STAT1-WT **(J)** or STAT1-MUT **(K)**. All values represent the mean ± s.d. of three independent experiments. *p < 0.05.

Using qRT-PCR, 13 predicted potential target genes were examined after mir-331-3p mimics were transfected into Marc-145 cells. As shown in Figure 3A, mir-331-3p significantly inhibited the expression of porcine *TNF-*α (*p* = 0.03), *TNFAIP1* (*p* = 0.011), *SOCS1* (*p* = 0.019), and *SCARA3* (*p* = 0.05) gene. Western blot analysis revealed that TNF-α protein expression levels were significantly suppressed by mir-331-3p mimics (Figure 3B), whereas inhibition of mir-331-3p led to the increased expression of TNF-α protein (Figure 3C). The dual-luciferase reporter system was used to analyze the interaction between mir-331-3p and *TNF-*α gene (Figure 3F). The result indicated that luciferase activity of TNF-α-WT (containing TNF-α-3′UTR) was significantly suppressed (*p* = 0.04, *p* = 0.016, Figure 3D) by mir-331-3p mimics. However, mir-331-3p had no significant effect on the fluorescence activity of TNF-α-MUT plasmid (Figure 3E).

### Mir-210 Directly Targeted Porcine *STAT1*

The 9 potential target genes of mir-210 were selected by bioinformatics and were quantitatively analyzed by qRT-PCR. The results showed that mir-210 could reduce the mRNA expression level of *STAT1* gene about 3 times (*p* = 0.012), and significantly inhibit the expression of *SCARA3* and *DIMT1* (*p* = 0.026, *p* = 0.018, Figure 3G). Additionally, Western blot assay revealed that mir-210 mimics significantly decreased the protein level of *STAT1* (Figure 3H), whereas mir-210 inhibitor significantly increased the protein level of *STAT1* (Figure 3I). To assess whether mir-210 directly targets *STAT1* gene (Figure 3F), mir-210 mimics and luciferase reporter plasmid STAT1-WT (containing a portion of the *STAT*1 3′UTR) were co-transfected into Marc-145 cells, the luciferase activity of *STAT*1-WT was significantly down-regulated (*p* = 0.01*, p* = 0.011, Figure 3J). No change was observed when the putative mir-210 binding sites were mutated (*STAT1*-MUT) (Figure 3K). These results confirmed that mir-210 could downregulate the expression of *STAT1* by directly targeting it.

### *STAT1* Promoted Expression of Inflammation-Associated Genes

**Figure 4:**
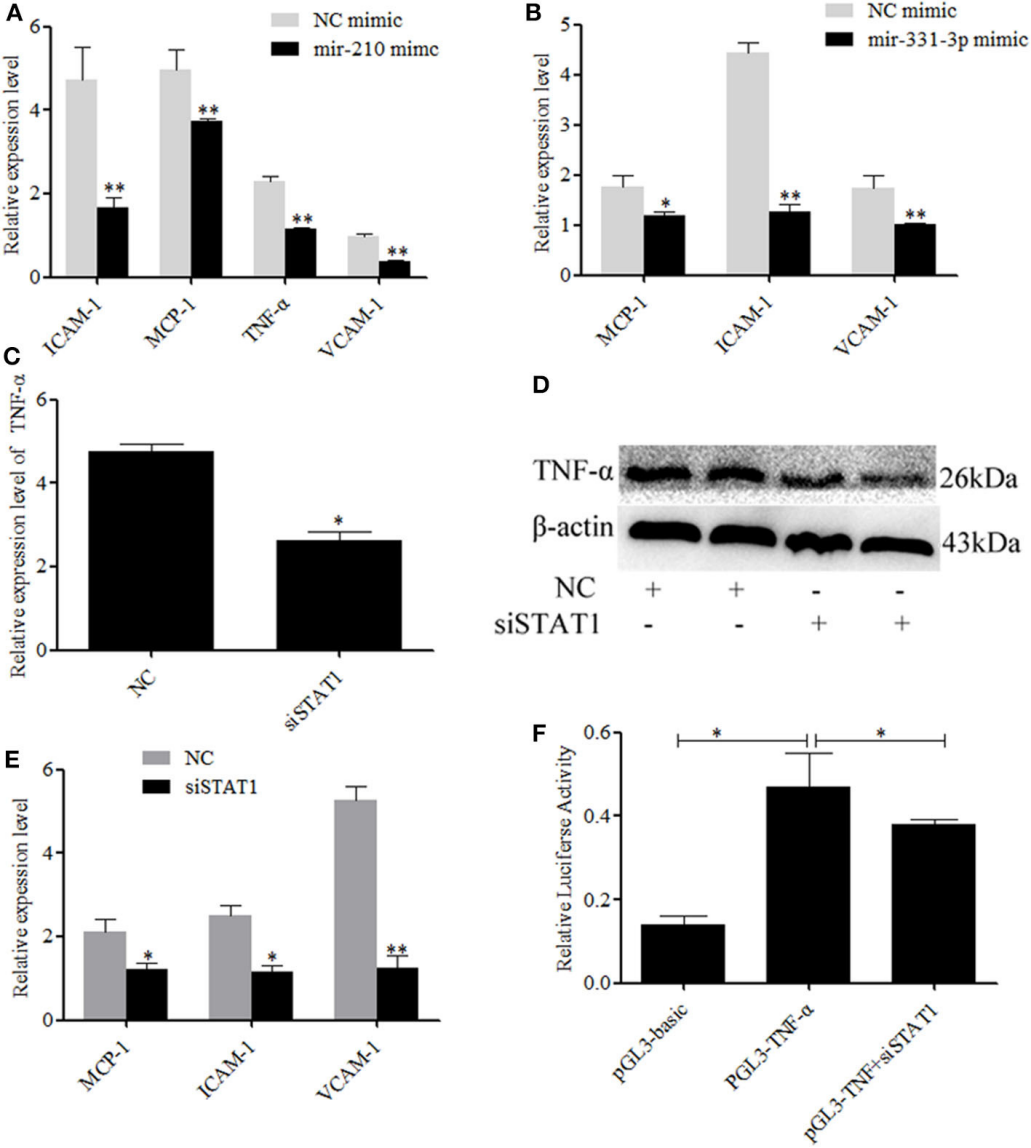
Mir-331-3p and mir-210 are involved in the regulation of genes that cause lung injury. Marc-145 cells were transfected with mir-210 mimics **(A)** or mir-331-3p mimics **(B)**, infected with the PRRSV-2 for 36 h, and the expressions of *MCP-1, ICAM-1*, and *VCAM-1* were detected by qRT-PCR. Knockdown of *STAT1* inhibited *TNF-*α mRNA expression **(C)** and protein expression **(D)**. Knockdown of *STAT1* also inhibited *MCP-1, ICAM-1*, and *VCAM-1* mRNA expression **(E)**. The siSTAT1 was co-transfected with PGL3-TNF-α into Marc-145 cells and reduced the luciferase activity **(F)**. All values represent the mean ± s.d. of three independent experiments. *p < 0.05, **p < 0.01.

Activate *STAT1* induces pro-inflammatory cytokine (36–38). Therefore, we investigated whether mir-210 and mir-331-3p affected the expression of inflammation-associated genes through *STAT1* and *TNF-*α. As expected, the expressions of intercellular adhesion molecule 1 (*ICAM-1*), macrophage cationic peptide 1 (*MCP-1*), and vascular cell adhesion molecule 1 (*VCAM-1*) were significantly inhibited by mir-210 (*p* = 0.01, *p* = 0.01, *p* = 0.0005, Figure 4A) or mir-331-3p (*p* = 0.0005, *p* = 0.049, *p* = 0.01, Figure 4B). Mir-210 also significantly inhibited the expression of *TNF-*α (*p* = 0.0004, Figure 4A), although bioinformatics analysis found that mir-210 was not able to directly bind *TNF-*α. To investigate whether *STAT1* gene has an effect on *TNF-*α in lung injury caused by PRRSV infection, si-STAT1 was transfected into Marc-145 cells. The results showed that si-STAT1 led to a significant decrease in *TNF-*α mRNA expression (*p* = 0.02, Figure 4C). Western blot analysis also revealed that *STAT1* promoted the expression of *TNF-*α (Figure 4D). The expression levels of *MCP-1, ICAM-1*, and *VCAM-1* were also significantly decreased after *STAT1* knockdown (*p* = 0.02, *p* = 0.01, *p* = 0.004, Figure 4E). To investigate whether *STAT1* directly acts on *TNF-*α, we analyzed the upstream sequence of *TNF-*α by using bioinformatics software. The results revealed that there is a binding site for *STAT1* in the upstream promoter region of *TNF-*α (Supplementary Figure 2). In addition, si-STAT1 significantly (*p* = 0.04) decreased the fluorescence activity of PGL3-TNF-α promoter (Figure 4F). These results suggested that *STAT1* promoted the expression of *TNF-*α by binding to the promoter region of *TNF-*α.

### Mir-331-3p Inhibited Viral Replication and Alleviated PRRSV-Induced Lung Injury *in vivo*

**Figure 5:**
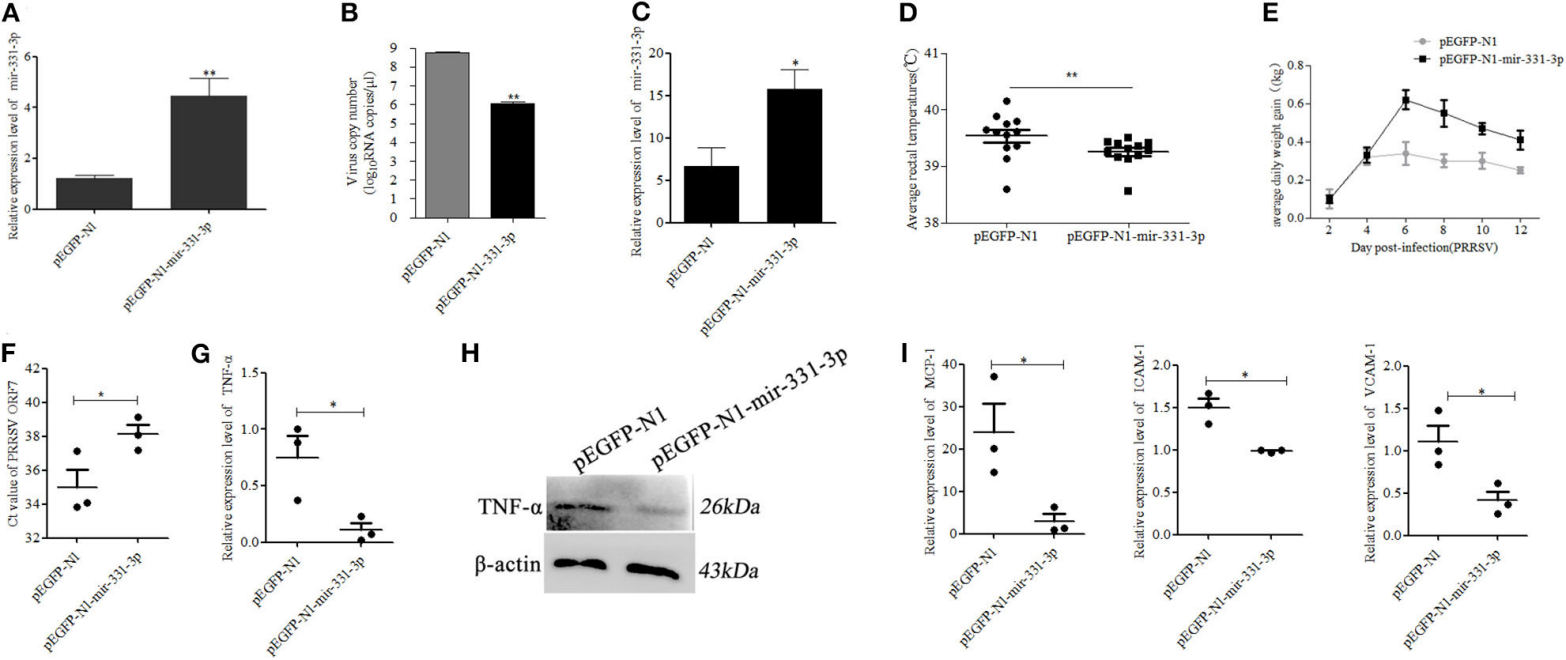
Mir-331-3p inhibits PRRSV-2 replication and lung injury *in vivo*. Marc-145 cells were transfected with pEGFP-N1-mir-331-3p or pEGFP-N1 (4 μg) to detect mir-331-3p expression **(A)** and the effect on PRRSV-2 replication **(B)**. The expression of mir-331-3p **(C)**, daily average rectal temperatures, **(D)** and average daily weight gain **(E)** of piglets infected PRRSV-2 after injection with plasmid pEGFP-N1 for the negative control group and pEGFP-N1-mir-331-3p for the experimental group. The Ct value of PRRSV ORF7 **(F)**, *TNF-*α mRNA expression **(G)**, TNF-α protein expression **(H)**, *MCP-1, VCAM-1*, and *ICAM-1* **(I)** mRNA expression was detected in the lungs of piglets from the experimental group and negative control group. All values represent the mean ± s.d. of three independent experiments. *p < 0.05, **p < 0.01.

To further confirm whether mir-331-3p had anti-inflammatory and antiviral effect, we constructed the overexpressed plasmid pEGFP-N1-mir-331-3p. Firstly, pEGFP-N1-mir-331-3p was transfected into Marc-145 cells, the expression level of mir-331-3p was significantly increased by nearly three times (*p* = 0.009, Figure 5A), and PRRSV-2 replication was dramatically inhibited by >90%, compared with the NC (*p* = 0.01, Figure 5B).

Then, six 4-week-old landrace piglets were divided into two groups (*n* = 3 per group). These piglets were infected with 1.5 mL 10^5.2^ TCID50 PRRSV-2 strain (WuH3) at 5 h post injection (2.5 mg/kg) of plasmid pEGFP-N1-mir-331-3p for the experimental group and pEGFP-N1 for the negative control group. The results of qRT-PCR showed that the expression of mir-331-3p was higher in the experimental group (*p* = 0.05, Figure 5C). The piglets were monitored for clinical signs, including anorexia, lethargy, fever, and weight. Two days after PRRSV-2 infection, pigs in the negative control group showed significant clinical symptoms such as abdominal breathing and increased rectal temperature, while the experimental group pigs presented clinical symptoms at 3 dpi. The average rectal temperature in the experimental group was significantly (*p* = 0.01) lower than that in the negative control group (Figure 5D). Average daily weight gain in piglets from the negative control group was lower compared with that from the experiment groups after PRRSV-2 infection (Figure 5E). At 14 dpi, all the piglets were euthanized and lungs were collected. The cycle threshold (Ct) of PRRSV ORF7 in lung tissues from the experimental group was found significantly (*p* = 0.05) higher than that from the negative control group (Figure 5F). The expression of the target gene TNF-α was also significantly (*p* = 0.05) inhibited by pEGFP-N1-mir-331-3p at both mRNA and protein level (Figures 5G,H). The expressions of inflammation-associated genes *MCP-1, ICAM-1*, and *VCAM-1* in experimental groups were also significantly decreased, compared with those in the negative control groups (*p* = 0.05, *p* = 0.05, *p* = 0.05, Figure 5I).

**Figure 6:**
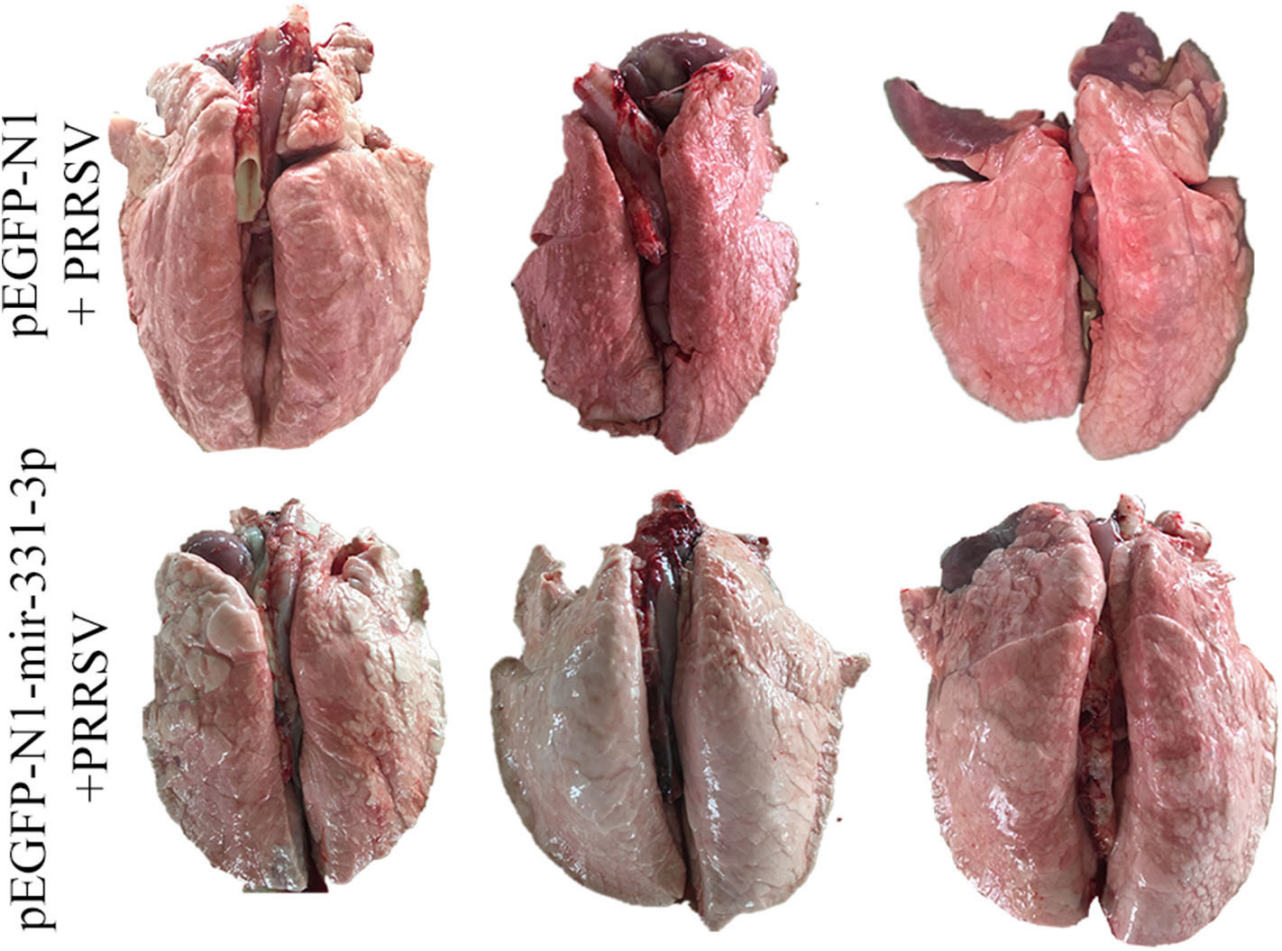
The pathological changes of lung surface of piglets from the experimental group and negative control group. After lung removal, the lung surface was rinsed with PBS, and then the lungs were placed on a white background plate and photographed. All six lungs are photographed at once.

**Figure 7:**
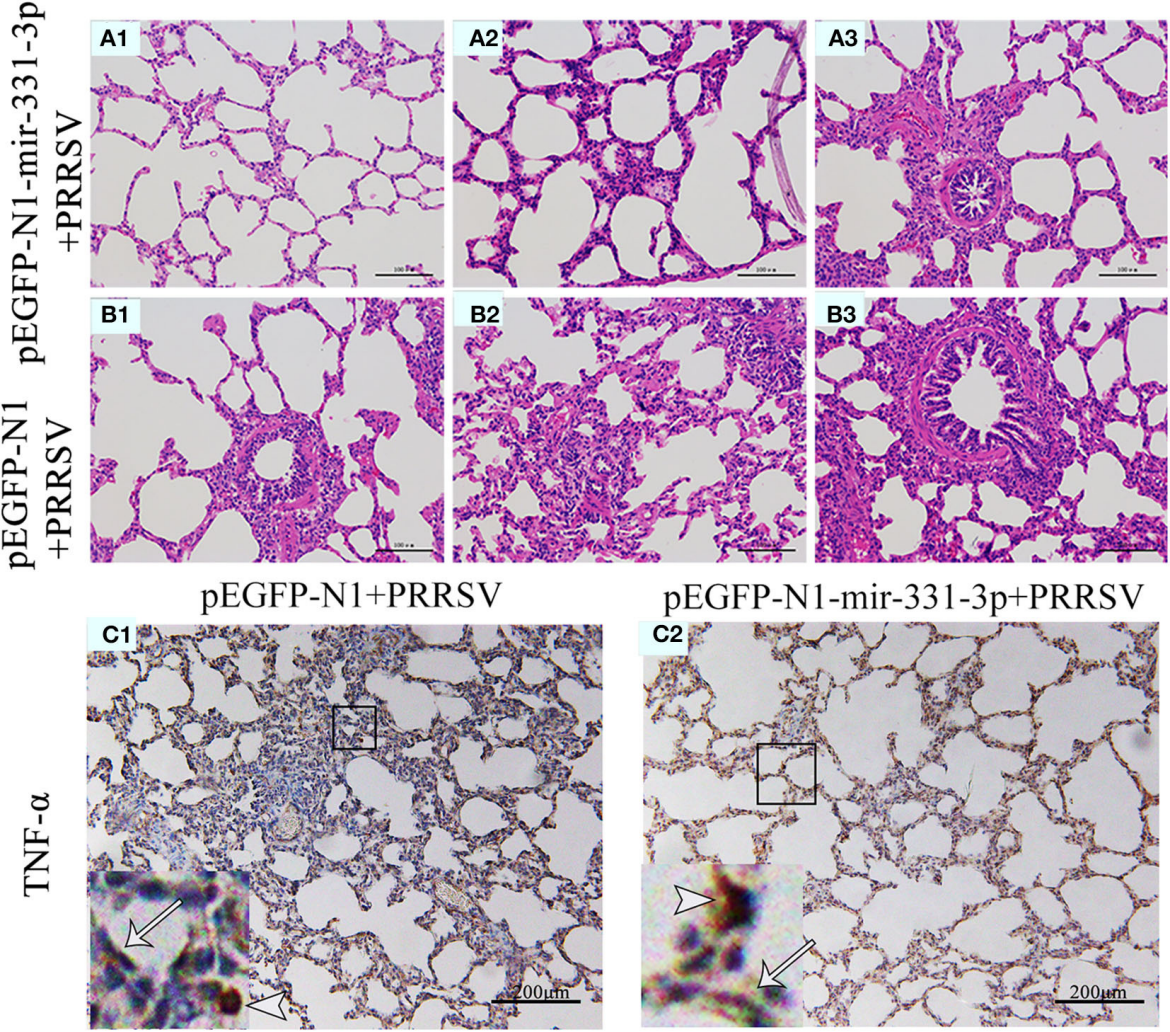
Histopathological and immunohistochemical analyses of lungs of piglet from the experimental group and negative control group. Histological sections of lungs of piglets from the experimental group injected with pEGFP-N1-mir-331-3p **(A1–A3)** and negative control group injected with pEGFP-N1 **(B1–B3)** were stained with hematoxylin-eosin. TNF-α **(C1,C2)** were immunohistochemically localized in porcine lungs from negative control group and the experimental group, respectively. TNF-positively stained cells are in reddish brown color. PAMs were labeled with short arrows, while alveolar epithelial cells were labeled with long arrows.

Afterwards, we also assessed the extent of macroscopic lung lesions and histopathological damage. As shown in Figure 6, the lungs of the negative control group were dark red due to congestion, while those of the experimental group were lighter in color. The interstitial pneumonia was more severe in the lungs of the negative control group than that of the experiment group. Hematoxylin-eosin staining showed that interstitial enlargement and congestion was more prominent in the negative control group than that in the experiment group (Figures 7A,B). Localization of TNF-α was detected in the lung tissues of pEGFP-N1-mir-331-3p group, pEGFP-N1 group by immunohistochemical staining. The result showed that TNF-α was mainly localized in lung epithelial cells and PAMs in the lung tissues of PRRSV-2 infected pigs (Figure 7C). The high expression of pro-inflammatory factor TNF-α induces lung injury. Mir-331-3p could inhibit the accumulation of TNF-α and lung injury.

## Discussion

Numerous studies have shown that the host miRNAs are involved in host-pathogen interactions and the regulation of immune responses and inflammation. Previous reports demonstrated that mir-181 inhibited viral replication by targeting PRRSV 3′ UTR (39), and that mir-30b-5p played an important role in lung injury in children (27). In our study, total cellular RNA was isolated from PRRSV-2 infected/mock-infected PAMs of Meishan, Landrace, Pietrain, and Qingping pigs at 9, 36, and 60 hpi to analyze the differential expression of miRNAs. To avoid the influence of mixed-leukocyte reactions (MLRs) on miRNA expression caused by mixed PAMs, an improved method was applied to increase the purity of the obtained PAMs (28, 29). In addition, PAMs from each animal have been infected by PRRSV before mixing. The unmixed PAMs were used in the qRT-PCR verification of differentially expressed miRNAs. Mir-331-3p was the only common DE-miR between PRRSV-infected and mock-infected PAMs of 4 pig breeds at 36 hpi and one of 4 common DE-miRs between PRRSV-infected and mock-infected PAMs of Meishan, Pietrain, and Landrace pigs at 60 hpi. Mir-210 was one of 7 common DE-miRs between PRRSV-infected and mock-infected PAMs of Meishan, Pietrain, and Qingping pigs at 60 hpi. It is consistent with the previous study that found mir-210 and mir-331 was differentially expressed in PBMCs from HIV-1–infected and uninfected individuals (40).

In addition, mir-331-3p and mir-210 were predicted to directly target PRRSV-2 ORF1b, and verified by double luciferase assay. ORF1b encoded multiple proteins that were further processed into multiple small protein products including Nsp9, Nsp10, Nsp11, and Nsp12, which were called non-structural proteins (Nsp). Of them, Nsp9 and Nsp10 were key enzymes for RNA synthesis of arterial virus, and closely related to the replication efficiency *in vitro* and *in vivo* and related to the increased pathogenicity and fatal virulence for piglets (12). Li et al. (41) showed that PRRSV-specific cytopathic effect (CPE) could be inhibited in the cells by shRNA targeting *ORF1b*, and that cellular virus titers were decreased by ~100-folds compared with those of control cells. Li et al. (42) reported that two recombinant adenoviruses expressing shRNA could effectively inhibit PRRSV replication *in vitro* and *in vivo* by targeting ORF1b of PRRSV. Our study also revealed mir-210 and mir-331-3p could both significantly inhibit PRRSV replication.

Moreover, bioinformatics analysis and experiment results confirmed that *TNF-*α is the target gene of mir-331-3p. It is consistent with a previous study that found mir-331-3p targeted TNF-α and notably weakened its expression in VSMC (43). TNF-α is a pleiotropic cytokine that mediates host response to infections and play decisive roles in the outcome of a number of viral infections, contributing to virus control or immune mediated pathology. TNF-α inhibitors have been successfully used in the clinic to treat these immune-mediated diseases (44, 45). TNF-α has also been implicated in a variety of pulmonary diseases and plays a crucial role in the occurrence and development of lung injury and fibrosis (46, 47). Gomez-Laguna et al. (48) reported the expression of TNF-α in the lungs of pigs infected with PRRSV-1 was correlated with the development of the interstitial pneumonia typical of this disease. Nukuzuma et al. (49) reported TNF-α stimulation could induce JC polyomavirus (JCV) replication through the NF-κB pathway in IMR-32 cells transfected with JCV DNA. Han et al. (15) reported that the pigs infected with HP-PRRSV showed the higher levels of TNF-α and exhibited severe pathological changes of lungs, which were in part responsible for the additional morbidity and mortality observed in HP-PRRSV infection (42). Sun et al. (50) reported that matrine possesses activity against PRRSV/PCV2 co-infection *in vitro* and suppression of the TLR3,4/NF-κB/TNF-α pathway as an important underlying molecular mechanism. Ge et al. (51) reported that PRRSV replication was suppressed in Marc-145 cells treated with EGCG post-infection, likely because of down-regulation of pro-inflammatory cytokines, such as TNF-α. Yang et al. (52) demonstrated that *TNF-*α might be a major contributor in ii/r-induced lung injury, and that the knockdown of *TNF-*α alleviated the severity of lung injury. Yu et al. (53) reported that downregulation of TNF-α signals by AT-Lipoxin A4 might be a significant mechanism in the attenuation in severe acute pancreatitis-associated lung injury. Our study also indicated that mir-331-3p inhibited the expression of *TNF-*α by directly targeting its 3′UTR. Mir-331-3p could attenuate lung injury and significantly inhibit viral replication by intramuscular injection of the expression plasmid of mir-331-3p *in vivo*. To further illuminate the mechanisms that underlie the impact of *TNF-*α on lung inflammation/injury, we also examined and found mir-331-3p suppressed the expression of inflammation-associated genes *MCP-1, VCAM-1*, and *ICAM-1 in vitro* and *in vivo*. On the other hand, down-regulation of TNF-a might be beneficial for the early phase of PRRSV infection, and it might also cause a prolongation of PRRSV infection. Activation of NF-κB signaling is one of the most important canonical responses to the stimulation of TNF-α (44). When NF-κB is activated, NF-κB will rapidly transfer from the cytoplasm to the nucleus, acting as transcription factor for several adhesion molecules and inflammatory cytokines, such as VCAM-1, ICAM-1, and MCP-1, which play vital roles in inflammatory diseases such as lung inflammation/injury (54–56). Thus, we inferred that mir-331-3p suppressed the expression of *MCP-1, VCAM-1*, and *ICAM-1* through inhibiting TNF-α-induced NF-κB activation. Of course, it needs further study.

Bioinformatics analysis and experiment results confirmed that *STAT1* is the target gene of mir-210. *STAT1* has been identified as a transcription factor, which is the important part of the cell signal pathway JAK/STAT, and plays a key role in lung injury and other inflammatory diseases. *STAT1* is positioned as the trigger for an entire set of immune-response genes with antiviral function (57). The activated *STAT1* (phosphorylation *STAT1*) is transported into the nucleus and then promotes the upregulation of pro-inflammatory factors including *TNF*-α, *MCP-1, VCAM-1*, and *ICAM-1* (58–61). *STAT1* antisense oligonucleotides (ASON) could inhibit the secretion of *TNF*-α and *ICAM-1* in alveolar macrophages (AMs), and *STAT1* could become a target of treating pulmonary fibrosis (62, 63). In our study, siSTAT1 also suppressed the expression of *TNF-*α, *MCP-1, VCAM-1*, and *ICAM-1*. Thus, we inferred that mir-210 suppressed the expression of *TNF-*α, *MCP-1, VCAM-1*, and *ICAM-1* through inhibiting *STAT1*-mediated transcriptional activation. Of course, it needs further study.

**Figure 8:**
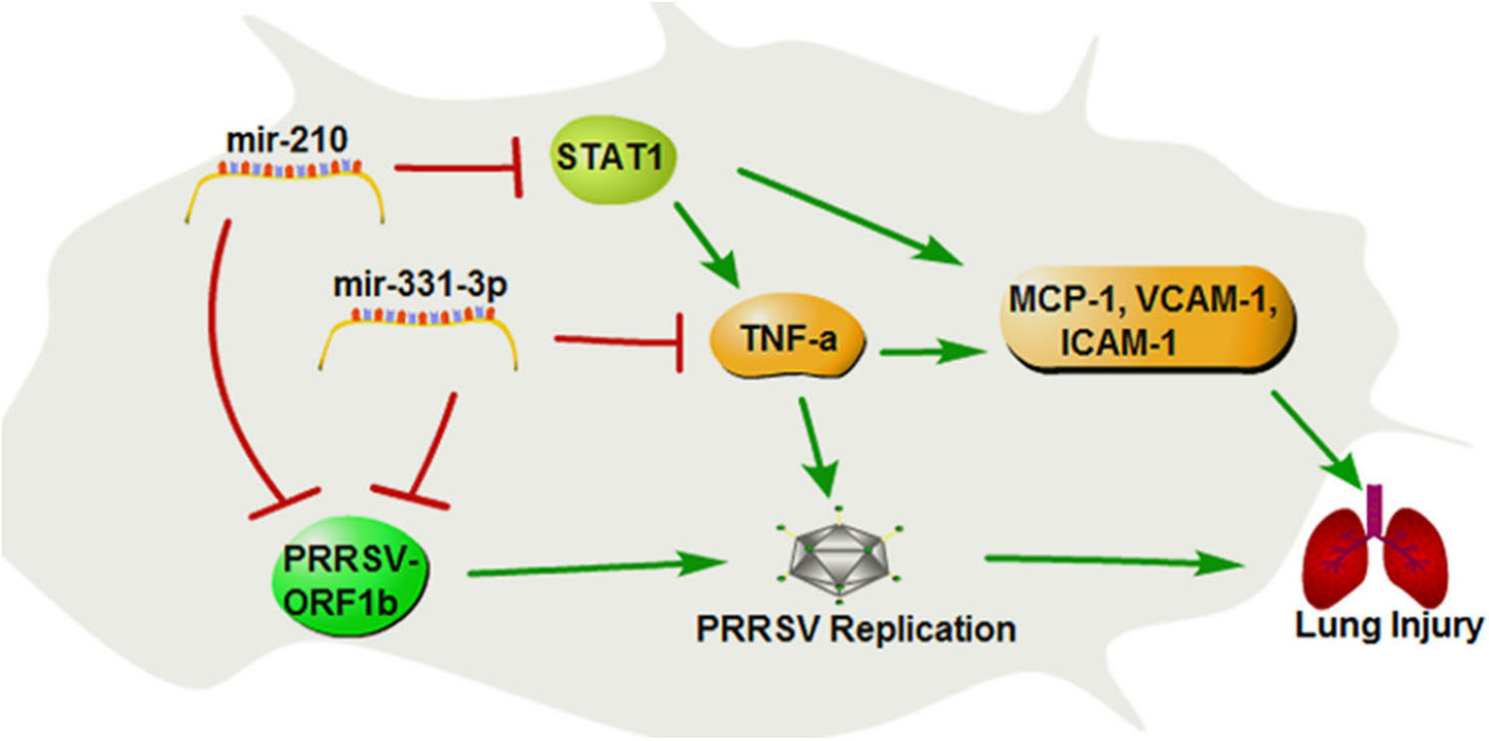
The regulation diagram of mir-210 and mir-331-3p inhibits PRRSV-2 replication and lung injury.

In summary, our study demonstrates that mir-331-3p and mir-210 could inhibit PRRSV-2 replication and lung injury by directly targeting PRRSV-2 ORF1b and porcine *TNF*-α and *STAT1*, which mediated the transcriptional activation of *MCP-1, VCAM-1*, and *ICAM-1*. *STAT1* could also upregulate the expression of *TNF-*α by binding to the promoter region of *TNF-*α (Figure 8). These insights may be applicable to PRRSV and helpful for the treatment of lung inflammation/injury.

## Data Availability Statement

The datasets presented in this study can be found in online repositories. The names of the repository/repositories and accession number(s) can be found in the article/Supplementary Material.

## Ethics Statement

The animal study was reviewed and approved by the Scientific Ethic Committee of Huazhong Agricultural University, Wuhan, China.

## Author Contributions

XY, YQ, YZ, XG, CH, GL, and QL carried out the experiments. XY, ML, DX, YQ, YZ, and JH analyzed the data. XY, ML, and DX wrote the manuscript. DX and ML designed the experiments and revised the manuscript. All authors contributed to the article and approved the submitted version.

## Conflict of Interest

The authors declare that the research was conducted in the absence of any commercial or financial relationships that could be construed as a potential conflict of interest.

## Supplementary Material

Supplementary Figure 1 Multi-strain comparison of the mir-210 or mir-331-3p at binding site sequences of PRRSV ORF1b.

Supplementary Figure 2 Bioinformatics analysis of TNF-α 5′flanking sequence. Bioinformatics prediction potential binding sites for *STAT1* in 5′flanking sequence of *TNF-*α **(A)**. The potential binding sites for *STAT1* in predicted promoter region of *TNF-*α **(B)**.

Supplementary Table 1 Sequences of primers used for plasmid construction in this study.

Supplementary Table 2 Sequences of miRNA mimics/inhibitors and siRNA used in this study.

Supplementary Table 3 Quantitative RT-PCR primer sequences used in this study.

## Abbreviations

PRRS: Porcine reproductive and respiratory syndrome
PRRSV: Porcine reproductive and respiratory syndrome virus
ORFs: Open reading frames
TNF-α: Tumor necrosis factor-α
STAT1: Signal transducer and activator of transcription 1
VCAM-1: Vascular adhesion molecule-1
ICAM-1: Intercellular adhesion molecule-1
MCP-1: Monocyte chemotactic protein-1
TNFAIP1: TNF alpha induced protein 1
SOCS1: Suppressor of cytokine signaling 1
SCARA3: Scavenger receptor class A member 3
DIMT1: DIMT1 rRNA methyltransferase and ribosome maturation factor
Nsp: non-structural proteins.

## References

[1] Terpstra C Wensvoort G Pol JMA Experimental reproduction of porcine epidemic abortion and respiratory syndrome (mystery swine disease) by infection with lelystad virus - koch postulates fulfilled (reprinted from the veterinary quarterly, Vol 13, Pg 131-136, 1991). Irish Vet J. (1993) 46:69–72. 10.1080/01652176.1991.9694297 1949539

[2] Nelsen CJ Murtaugh MP Faaberg KS. Porcine reproductive and respiratory syndrome virus comparison: divergent evolution on two continents. J Virol. (1999) 73:270-80. 10.1128/JVI.73.1.270-280.1999 9847330 PMC103831

[3] Wensvoort G Terpstra C Pol JMA Terlaak EA Bloemraad M Dekluyver EP. Mystery swine disease in the netherlands - the isolation of lelystad virus. Vet Q. (1991) 13:121–30. 10.1080/01652176.1991.9694296 1835211

[4] Collins JE Benfield DA Christianson WT Harris L Hennings JC Shaw DP. Isolation of swine infertility and respiratory syndrome virus (Isolate atcc vr-2332) in north-america and experimental reproduction of the disease in gnotobiotic pigs. J Vet Diagn Invest. (1992) 4:117–26. 10.1177/104063879200400201 1616975

[5] Charoenchanikran P Kedkovid R Sirisereewan C Woonwong Y Arunorat J Sitthichareonchai P. Efficacy of fosteraa (R) PRRS modified live virus (MLV) vaccination strategy against a Thai highly pathogenic porcine reproductive and respiratory syndrome virus (HP-PRRSV) infection. Trop Anim Health Prod. (2016) 48:1351–9. 10.1007/s11250-016-1099-1 27315207

[6] Tian KG Yu XL Zhao TZ Feng YJ Cao Z Wang CB. Emergence of fatal PRRSV variants: unparalleled outbreaks of atypical PRRS in china and molecular dissection of the unique hallmark. PLoS ONE. (2007) 2:e0000526. 10.1371/journal.pone.0000526 17565379 PMC1885284

[7] Guo ZH Chen XX Li R Qiao SL Zhang GP. The prevalent status and genetic diversity of porcine reproductive and respiratory syndrome virus in China: a molecular epidemiological perspective. Virol J. (2018) 15:2. 10.1186/s12985-017-0910-6 29301547 PMC5753475

[8] Lee HS Pham TL Nguyen TN Lee M Wieland B. Seasonal patterns and space-time clustering of porcine reproductive and respiratory syndrome (PRRS) cases from 2008 to 2016 in Vietnam. Transb Emerg Dis. (2019) 66:986–94. 10.1111/tbed.13122 30636103 PMC6850339

[9] Rowland RRR Joan L Jack D. Control of porcine reproductive and respiratory syndrome (PRRS)through genetic improvements in disease resistance and tolerance. Front Genet. (2012) 3:260 10.3389/fgene.2012.00260 23403935 PMC3565991

[10] Tang YD Fang QQ Liu JT Wang TY Wang Y Tao Y. Open reading frames 1a and 1b of the porcine reproductive and respiratory syndrome virus (PRRSV) collaboratively initiate viral minus-strand RNA synthesis. Biochem Biophys Res Commun. (2016) 477:927–31. 10.1016/j.bbrc.2016.06.161 27378424

[11] Bautista EM Faaberg KS Mickelson D McGruder ED. Functional properties of the predicted helicase of porcine reproductive and respiratory syndrome virus. Virology. (2002) 298:258–70. 10.1006/viro.2002.1495 12127789 PMC7130902

[12] Li Y Zhou L Zhang J Ge X Zhou R Zheng H. Nsp9 and Nsp10 contribute to the fatal virulence of highly pathogenic porcine reproductive and respiratory syndrome virus emerging in China. PLoS Pathog. (2014) 10:e1004216. 10.1371/journal.ppat.1004216 24992286 PMC4081738

[13] Lawson SR Rossow KD Collins JE Benfield DA Rowland RRR. Porcine reproductive and respiratory syndrome virus infection of gnotobiotic pigs: sites of virus replication and co-localization with MAC-387 staining at 21 days post-infection. Virus Res. (1997) 51:105–13. 10.1016/S0168-1702(97)00086-5 9498609

[14] Molitor TW Bautista EM Choi CS. Immunity to PRRSV: double-edged sword. Vet Microbiol. (1997) 55:265–76. 10.1016/S0378-1135(96)01327-2 9220622 PMC7117121

[15] Han D Hu Y Li L Tian H Chen Z Wang L. Highly pathogenic porcine reproductive and respiratory syndrome virus infection results in acute lung injury of the infected pigs. Vet Microbiol. (2014) 169:135–46. 10.1016/j.vetmic.2013.12.022 24472226 PMC7127595

[16] Wills RW Doster AR Galeota JA Sur JH Osorio FA. Duration of infection and proportion of pigs persistently infected with porcine reproductive and respiratory syndrome virus. J Clin Microbiol. (2003) 41:58–62. 10.1128/JCM.41.1.58-62.2003 12517825 PMC149563

[17] Jenne CN Wong CH Zemp FJ McDonald B Rahman MM Forsyth PA. Neutrophils recruited to sites of infection protect from virus challenge by releasing neutrophil extracellular traps. Cell Host Microbe. (2013) 13:169–80. 10.1016/j.chom.2013.01.005 23414757

[18] Grommes J Soehnlein O. Contribution of neutrophils to acute lung injury. Mol Med. (2011) 17:293–307. 10.2119/molmed.2010.00138 21046059 PMC3060975

[19] Arneth B. Systemic lupus erythematosus and DNA degradation and elimination defects. Front Immunol. (2019) 10:1697. 10.3389/fimmu.2019.01697 31440232 PMC6692764

[20] Chew GL Pauli A Schier AF. Conservation of uORF repressiveness and sequence features in mouse, human and zebrafish. Nat Commun. (2016) 7:11663. 10.1038/ncomms11663 27216465 PMC4890304

[21] Brar GA Yassour M Friedman N Regev A Ingolia NT Weissman JS. High-resolution view of the yeast meiotic program revealed by ribosome profiling. Science. (2012) 335:552–7. 10.1126/science.1215110 22194413 PMC3414261

[22] Staedel C Darfeuille F. MicroRNAs and bacterial infection. Cell Microbiol. (2013) 15:1496–507. 10.1111/cmi.12159 23795564

[23] Shi LY Zheng XQ Fan YZ Yang XL Li AM Qian J. The contribution of miR-122 to the innate immunity by regulating toll-like receptor 4 in hepatoma cells. BMC Gastroenterol. (2019) 19:1048. 10.1186/s12876-019-1048-3 31340754 PMC6657172

[24] Zhang FM Sun XF Zhu Y Qin WS. Downregulation of miR-146a inhibits influenza A virus replication by enhancing the type I interferon response *in vitro* and *in vivo*. Biomed Pharmacother. (2019) 111:740–50. 10.1016/j.biopha.2018.12.103 30611999

[25] Lin X Xu LY Li XH Yu TX Lin Q Chen L. Effect and mechanism of MicroRNA-146a on TLR4 inflammatory signal pathway in the lung tissues of rats with mechanical ventilator-induced lung injury. Zhonghua Yi Xue Za Zhi. (2018) 98:2749–53. 10.3760/cma.j.issn.0376-2491.2018.34.014 30220173

[26] Wang YF Zhang XR Tian JM Liu GZ Li XF Shen D. Sevoflurane alleviates LPS-induced acute lung injury via the microRNA-27a-3p/TLR4/MyD88/NF-kappa B signaling pathway. Int J Mol Med. (2019) 44:479–90. 10.3892/ijmm.2019.4217 31173183 PMC6605322

[27] Zhou T Chen YL. The functional mechanisms of miR-30b-5p in acute lung injury in children. Med Sci Monit. (2019) 25:40–51. 10.12659/MSM.911398 30600796 PMC6327783

[28] Meng C Su L Li Y Zhu Q Li J Wang H. Different susceptibility to porcine reproductive andrespiratory syndrome virus infection among Chinese native pig breeds. Arch Virol. (2018) 575 163:2155–64. 10.1007/s00705-018-3821-y 29691704

[29] Meng C Cao S Su L He Q Wang H Fen X Isolation, purification and cryopreservation ofporcine alveolar macrophages. Jiangsu Agric Sci. (2012) 40:198–201. 10.3969/j.issn.1002-1302.2012.11.081

[30] Li G Li Y Li X Ning X Li M Yang G. MicroRNA identity and abundance in developing swine adipose tissue as determined by solexa sequencing. J Cell Biochem. (2011) 112:1318–28. 10.1002/jcb.23045 21312241

[31] Wang Y Zhang C Fang X Zhao Y Chen X Sun J. Identification and profiling of microRNAs and their target genes from developing caprine skeletal muscle. PLoS ONE. (2014) 9:e96857. 10.1371/journal.pone.0096857 24818606 PMC4018397

[32] Ding H Liu M Zhou C You X Suo Z Zhang C. Expression and regulation of GnRHR2 gene and testosterone secretion mediated by GnRH2 and GnRHR2 within porcine testes. J Steroid Biochem Mol Biol. (2019) 190:161–72. 10.1016/j.jsbmb.2019.03.010 30930217

[33] Bordet E Blanc F Tiret M Crisci E Bouguyon E Renson P. Porcine reproductive and respiratory syndrome virus type 1.3 lena triggers conventional dendritic cells 1 activation and t helper 1 immune response without infecting dendritic cells. Front Immunol. (2018) 9:2299. 10.3389/fimmu.2018.02299 30333837 PMC6176214

[34] Bordet E Fretaud M Crisci E Bouguyon E Rault S Pezant J. Macrophage-B cell interactions in the inverted porcine lymph node and their response to porcine reproductive and respiratory syndrome virus. Front Immunol. (2019) 10:953. 10.3389/fimmu.2019.00953 31130951 PMC6510060

[35] Skreka K Schafferer S Nat IR Zywicki M Salti A Apostolova G. Identification of differentially expressed non-coding RNAs in embryonic stem cell neural differentiation. Nucleic Acids Res. (2012) 40:6001–15. 10.1093/nar/gks311 22492625 PMC3401476

[36] Kou X Qi S Dai W Luo L Yin Z. Arctigenin inhibits lipopolysaccharide-induced iNOS expression in RAW264.7 cells through suppressing JAK-STAT signal pathway. Int Immunopharmacol. (2011) 11:1095–102. 10.1016/j.intimp.2011.03.005 21426947

[37] Hoyo-Becerra C Huebener A Trippler M Lutterbeck M Liu ZJ Truebner K. Concomitant interferon alpha stimulation and TLR3 activation induces neuronal expression of depression-related genes that are elevated in the brain of suicidal persons. PLoS ONE. (2013) 8:e83149. 10.1371/journal.pone.0083149 24391741 PMC3877033

[38] Yu Y Wang R Nan Y Zhang L Zhang Y. Induction of STAT1 phosphorylation at serine 727 and expression of proinflammatory cytokines by porcine reproductive and respiratory syndrome virus. PLoS ONE. (2013) 8:e61967. 10.1371/journal.pone.0061967 23637938 PMC3634824

[39] Guo XK Zhang Q Gao L Li N Chen XX Feng WH. Increasing expression of microRNA 181 inhibits porcine reproductive and respiratory syndrome virus replication and has implications for controlling virus infection. J Virol. (2013) 87:1159–71. 10.1128/JVI.02386-12 23152505 PMC3554091

[40] Ballegaard V Ralfkiaer U Pedersen KK Hove M Koplev S Braendstrup P. MicroRNA-210, microRNA-331, and microRNA-7 are differentially regulated in treated HIV-1-infected individuals and are associated with markers of systemic inflammation. J Acquir Immune Defic Syndr. (2017) 74:e104–13. 10.1097/QAI.0000000000001191 27749601

[41] Li G Huang J Jiang P Li Y Jiang W Wang X. Suppression of porcine reproductive and respiratory syndrome virus replication in MARC-145 cells by shRNA targeting ORF1 region. Virus Genes. (2007) 35:673–9. 10.1007/s11262-007-0134-8 17671836

[42] Li GM Jiang P Li YF Wang XW Huang J Bai J. Inhibition of porcine reproductive and respiratory syndrome virus replication by adenovirus-mediated RNA interference both in porcine alveolar macrophages and swine. Antivir Res. (2009) 82:157–65. 10.1016/j.antiviral.2009.02.202 19428607

[43] Fan W Liu Y Li C Qu X Zheng G Zhang Q. microRNA-331-3p maintains the contractile type of vascular smooth muscle cells by regulating TNF-alpha and CD14 in intracranial aneurysm. Neuropharmacology. (2020) 164:107858. 10.1016/j.neuropharm.2019.107858 31785262

[44] Wang W Xu L Brandsma JH Wang Y Hakim MS Zhou X. Convergent Transcription of Interferon-stimulated genes by TNF-alpha and IFN-alpha augments antiviral activity against HCV and HEV. Sci Rep. (2016) 6:25482. 10.1038/srep25482 27150018 PMC4858707

[45] Amarilla SP Gomez-Laguna J Carrasco L Rodriguez-Gomez IM Caridad YOJM Morgan SB. A comparative study of the local cytokine response in the lungs of pigs experimentally infected with different PRRSV-1 strains: upregulation of IL-1alpha in highly pathogenic strain induced lesions. Vet Immunol Immunopathol. (2015) 164:137–47. 10.1016/j.vetimm.2015.02.003 25739319

[46] Narita K Kuwabara Y Fujii Y. Lung injury after intestinal ischemia-reperfusion may be avoided by the reduced absorption of locally produced cytokines. Surg Today. (2004) 34:937–42. 10.1007/s00595-004-2847-9 15526129

[47] Caty MG Guice KS Oldham KT Remick DG Kunkel SI. Evidence for tumor necrosis factor-induced pulmonary microvascular injury after intestinal ischemia-reperfusion injury. Ann Surg. (1990) 212:694–700. 10.1097/00000658-199012000-00007 2175168 PMC1358254

[48] Gomez-Laguna J Salguero FJ Barranco I Pallares FJ Rodriguez-Gomez IM Bernabe A. Cytokine expression by macrophages in the lung of pigs infected with the porcine reproductive and respiratory syndrome virus. J Comp Pathol. (2010) 142:51–60. 10.1016/j.jcpa.2009.07.004 19691969 PMC7126906

[49] Nukuzuma S Nakamichi K Kameoka M Sugiura S Nukuzuma C Tasaki T. TNF-alpha stimulates efficient JC virus replication in neuroblastoma cells. J Med Virol. (2014) 86:2026–32. 10.1002/jmv.23886 24415534

[50] Sun N Sun P Lv H Sun Y Guo J Wang Z. Matrine displayed antiviral activity in porcine alveolar macrophages co-infected by porcine reproductive and respiratory syndrome virus and porcine circovirus type 2. Sci Rep. (2016) 6:24401. 10.1038/srep24401 27080155 PMC4832146

[51] Ge M Xiao Y Chen H Luo F Du G Zeng F. Multiple antiviral approaches of (-)-epigallocatechin-3-gallate (EGCG) against porcine reproductive and respiratory syndrome virus infection in vitro. Antiviral Res. (2018) 158:52–62. 10.1016/j.antiviral.2018.07.012 30048655

[52] Yang Z Zhang XR Zhao Q Wang SL Xiong LL Zhang P. Knockdown of TNF-alpha alleviates acute lung injury in rats with intestinal ischemia and reperfusion injury by upregulating IL-10 expression. Int J Mol Med. (2018) 42:926–34. 10.3892/ijmm.2018.3674 29767265 PMC6034932

[53] Yu SH Xie JM Xiang YK Dai SJ Yu DL Sun HW. Downregulation of TNF-/TNF-R1 Signals by AT-Lipoxin A4 may be a significant mechanism of attenuation in SAP-associated lung injury. Mediat Inflamm. (2019) 2019:9019404. 10.1155/2019/9019404 31097921 PMC6487108

[54] Huang W Huang M Ouyang H Peng J Liang J. Oridonin inhibits vascular inflammation by blocking NF-kappaB and MAPK activation. Eur J Pharmacol. (2018) 826:133–9. 10.1016/j.ejphar.2018.02.044 29518395

[55] Liang J Yuan S Wang X Lei Y Zhang X Huang M. Attenuation of pristimerin on TNF-alpha-induced endothelial inflammation. Int Immunopharmacol. (2020) 82:106326. 10.1016/j.intimp.2020.106326 32135490

[56] Liu W Dong M Bo L Li C Liu Q Li Z. Epigallocatechin-3-gallate suppresses alveolar epithelial cell apoptosis in seawater aspiration-induced acute lung injury via inhibiting STAT1-caspase-3/p21 associated pathway. Mol Med Rep. (2016) 13:829–36. 10.3892/mmr.2015.4617 26647880

[57] Look DC Roswit WT Frick AG Gris-Alevy Y Dickhaus DM Walter MJ. Direct suppression of Stat1 function during adenoviral infection. Immunity. (1998) 9:871–80. 10.1016/S1074-7613(00)80652-4 9881977

[58] Stojanovic T Wagner AH Wang S Kiss E Rockstroh N Bedke J. STAT-1 decoy oligodeoxynucleotide inhibition of acute rejection in mouse heart transplants. Basic Res Cardiol. (2009) 104:719–29. 10.1007/s00395-009-0028-0 19352584 PMC3085763

[59] Lee YW Hennig B Toborek M. Redox-regulated mechanisms of IL-4-induced MCP-1 expression in human vascular endothelial cells. Am J Physiol Heart Circul Physiol. (2003) 284:H185–92. 10.1152/ajpheart.00524.2002 12388243

[60] Darnell JE Jr. STATs and gene regulation. Science. (1997) 277:1630–5. 10.1126/science.277.5332.1630 9287210

[61] Liu W Dong M Bo L Li C Liu Q Li Y. Epigallocatechin-3-gallate ameliorates seawater aspiration-induced acute lung injury via regulating inflammatory cytokines and inhibiting JAK/STAT1 pathway in rats. Mediat Inflamm. (2014) 2014:612593. 10.1155/2014/612593 24692852 PMC3945896

[62] Zeng M Liao B Zhu C Wang WJ Zhan XQ Fan XM. Aerosolized STAT1 antisense oligodeoxynucleotides decrease the concentrations of inflammatory mediators in bronchoalveolar lavage fluid in bleomycin-induced rat pulmonary fibrosis. Cell Mol Immunol. (2008) 5:219–24. 10.1038/cmi.2008.27 18582404 PMC4651273

[63] Fan X Wang Z. STAT1 antisense oligonucleotides attenuate the proinflammatory cytokine release of alveolar macrophages in bleomycin-induced fibrosis. Cell Mol Immunol. (2005) 2:211–7. 16212889

