# Supplementary figures and images for: Mir-331-3p Inhibits PRRSV-2 Replication and Lung Injury by Targeting PRRSV-2 ORF1b and Porcine TNF-α
Source: Front Immunol. 2020 Sep 25;11:547144. doi: 10.3389/fimmu.2020.547144 (PMC7544944; doi:10.3389/fimmu.2020.547144)

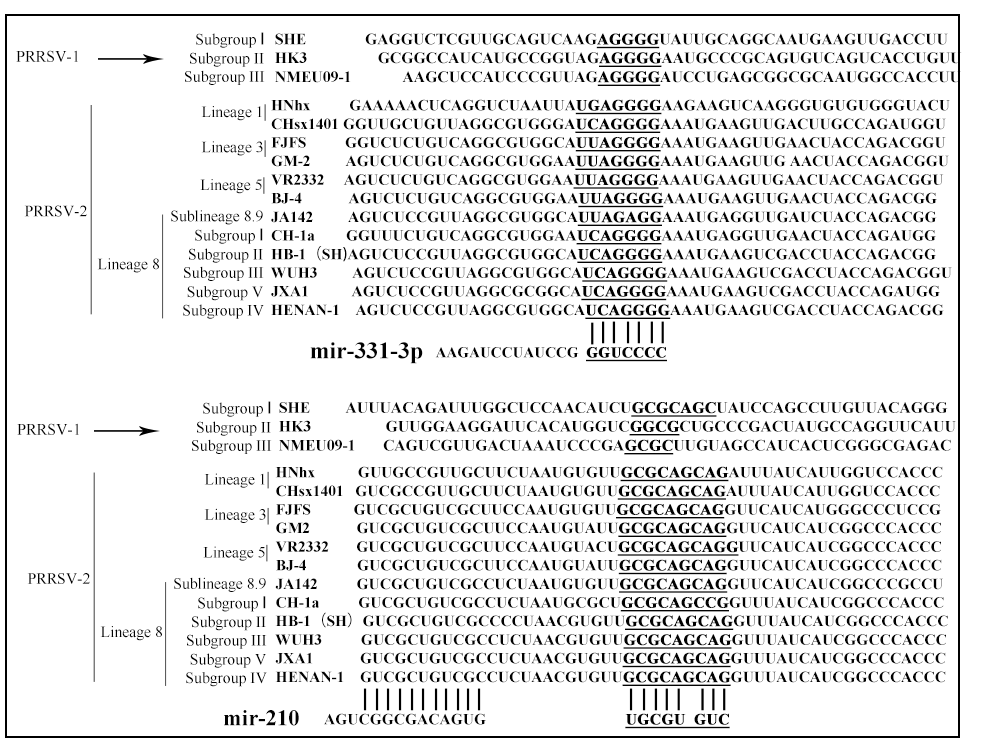

Supplement: Supplementary Figure 1 — Multi-strain comparison of the mir-210 or mir-331-3p at binding site sequences of PRRSV ORF1b. [file Image_1.TIF]

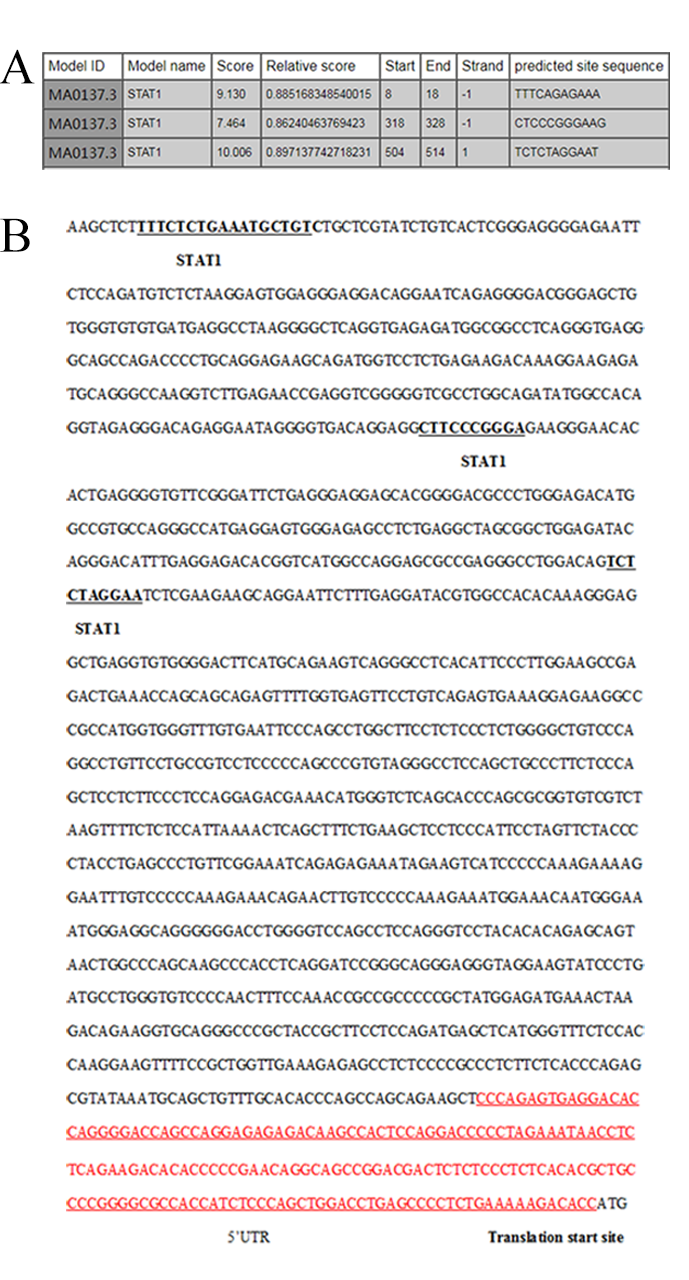

Supplement: Supplementary Figure 2 — Bioinformatics analysis of TNF-α 5′flanking sequence. Bioinformatics prediction potential binding sites for STAT1 in 5′flanking sequence of TNF-α (A). The potential binding sites for STAT1 in predicted promoter region of TNF-α (B). [file Image_2.TIF]
